# Exposure of Bacterial Biofilms to Electrical Current Leads to Cell Death Mediated in Part by Reactive Oxygen Species

**DOI:** 10.1371/journal.pone.0168595

**Published:** 2016-12-19

**Authors:** Cassandra L. Brinkman, Suzannah M. Schmidt-Malan, Melissa J. Karau, Kerryl Greenwood-Quaintance, Daniel J. Hassett, Jayawant N. Mandrekar, Robin Patel

**Affiliations:** 1 Division of Clinical Microbiology, Department of Laboratory Medicine and Pathology, Mayo Clinic, Rochester, MN, United States of America; 2 Department of Molecular Genetics, Biochemistry & Microbiology, University of Cincinnati College of Medicine, Cincinnati, OH, United States of America; 3 Division of Biomedical Statistics and Informatics, Department of Health Sciences Research Mayo Clinic, Rochester, MN, United States of America; 4 Division of Infectious Diseases, Department of Medicine, Mayo Clinic, Rochester, MN, United States of America; Universiteit Gent, BELGIUM

## Abstract

Bacterial biofilms may form on indwelling medical devices such as prosthetic joints, heart valves and catheters, causing challenging-to-treat infections. We have previously described the ‘electricidal effect’, in which bacterial biofilms are decreased following exposure to direct electrical current. Herein, we sought to determine if the decreased bacterial quantities are due to detachment of biofilms or cell death and to investigate the role that reactive oxygen species (ROS) play in the observed effect. Using confocal and electron microscopy and flow cytometry, we found that direct current (DC) leads to cell death and changes in the architecture of biofilms formed by Gram-positive and Gram-negative bacteria. Reactive oxygen species (ROS) appear to play a role in DC-associated cell death, as there was an increase in ROS-production by *Staphylococcus aureus* and *Staphylococcus epidermidis* biofilms following exposure to DC. An increase in the production of ROS response enzymes catalase and superoxide dismutase (SOD) was observed for *S*. *aureus*, *S*. *epidermidis* and *Pseudomonas aeruginosa* biofilms following exposure to DC. Additionally, biofilms were protected from cell death when supplemented with antioxidants and oxidant scavengers, including catalase, mannitol and Tempol. Knocking out SOD (*sodAB*) in *P*. *aeruginosa* led to an enhanced DC effect. Microarray analysis of *P*. *aeruginosa* PAO1 showed transcriptional changes in genes related to the stress response and cell death. In conclusion, the electricidal effect results in death of bacteria in biofilms, mediated, at least in part, by production of ROS.

## Introduction

Although the formation of bacterial biofilms has been appreciated for some time, serious investigations and advances in knowledge and mechanisms regarding bacterial biofilm formation, resistance to antibiotics/biocides and dispersal have only been realized over the last two decades [1]. Biofilms are the most common means by which bacteria associate with one another in nature and are important in clinical medicine [2]. The prevalence of biofilm-associated infections is partly a result of the widespread use of medical devices [1]. According to the Centers for Disease Control, in 2007, there were approximately 1.7 million hospital-acquired infections attributable to bacterial biofilms, leading to an economic burden of $11 billion [1]. The annual cost of biofilm-associated infections is estimated to be $94 billion and is responsible for over a half a million deaths [3]. Infections caused by bacterial biofilms include chronic lung infections caused by *Pseudomonas aeruginosa* in patients with cystic fibrosis and wound infections [1]. Additionally, biofilm-related infections are found on indwelling medical devices, such as urinary catheters, central lines, left ventricular assist device drive lines, heart valves, and prosthetic joints [2, 4]. Microbial biofilms associated with clinical infections provide a protective environment in which bacteria are 10–100 times more resistant to antimicrobial agents compared to their planktonic counterparts, hampering treatment options [5].

The use of electricity to break down and destroy bacterial biofilms has been investigated for a number of years [6–9]. Our interest in this phenomenon began when we investigated the effects of various antimicrobial treatments paired with electrical current, and observed that treatment with direct current (DC) alone (200 μA) led to significant decreases in *Staphylococcus aureus*, *Staphylococcus epidermidis* and *P*. *aeruginosa* biofilms [10]. Next, preliminary experiments were conducted to determine if this “electricidal effect” (the name we proposed for the observed effect) was active *in vivo* [11]. We found that rabbits with experimental *S*. *epidermidis* foreign body osteomyelitis exposed to 200 μA DC for 21 days had a decrease in bacterial quantities compared to those that were not exposed to current [11]. To date, we have investigated 33 different bacterial and fungal strains, representing 13 species of microorganisms, and have observed a time and dose-dependent electricidal effect against most isolates tested [12]. We have also demonstrated activity of lower amounts of DC (2, 5 or 10 μA) against most isolates tested [12]. Additionally, we have found that the electrical current does not need to be continuously applied; for example, application of 200 μA DC for as little as 2 hours per day over a 4 day period reduces biofilms [12]. The next logical step was to determine if the electricidal effect promotes biofilm detachment and/or leads to cell death and to also elucidate the factors that play a role in the observed effect.

Reactive oxygen species (ROS), including superoxide (O_2_^-^), hydrogen peroxide (H_2_O_2_), hydroxyl radical (HO^.^), and other oxygen- or nitrogen-based reactive species, are an expected byproduct of cells undergoing normal aerobic respiration. ROS damage lipids, proteins, RNA, DNA and critical associated cofactors, resulting in harm which, if severe enough, leads to widespread cellular damage and eventually cell death [13]. To prevent these effects, bacterial cells produce enzymes (e.g., catalase, peroxidase, superoxide dismutase [SOD], and alkylhydroperoxide reductase [14]) that break down toxic oxygen species [13]. Over the past decade, it has been shown that there may be a beneficial role of low concentrations of ROS in bacteria [15]. ROS are, however, only helpful to bacteria when stress to the cell is low and transient. When cellular stress increases and is persistent, increasing levels of ROS overwhelm the protective effects of the aforementioned enzymes, leading to cellular damage and death [15, 16]. In studies investigating migration of glioma cells, application of DC induced production of ROS which, in turn, influenced cell migration [17]. We hypothesize that the decrease in bacterial biofilms following exposure to DC is, in part, due to the over-production of damaging ROS [6].

Lipid peroxidation is also an effect of ROS, as a result of damage to lipid membranes; this can lead to loss of membrane integrity. Hydroxyl radicals (HO^.^), produced by a Fenton-like reaction, drive nonenzymatic peroxidation of unsaturated fatty acids, inducing a series of reactions that lead to structural changes of the lipid bilayer, resulting in loss of membrane integrity [18]. The detection of lipid peroxidation is therefore a marker of oxidative stress.

The manner by which decreases in bacterial quantities are achieved by the application of DC to bacterial biofilms is incompletely defined. Herein, we report results of studies to determine if cell death or detachment is responsible for decreased bacterial quantities and we assess the role of ROS and associated enzymes in cell death following exposure of bacterial biofilms to DC. We also report the effects of DC on biofilms formed by a *P*. *aeruginosa* mutant devoid of SOD, and the effects of DC on lipid peroxidation and transcriptional changes in response to exposure *P*. *aeruginosa* biofilms to DC.

## Materials and Methods

### Microorganisms

*S*. *epidermidis* Xen 43, *P*. *aeruginosa* Xen 5, *P*. *aeruginosa* PAO1, *P*. *aeruginosa* Δ*sodAB*, *S*. *aureus* Xen 30, were studied. The Xen strains were generous gifts of Perkin Elmer Caliper Life Sciences formerly known as Xenogen Corp., Waltham, MA. PAO1 and PAO1Δ*sodAB* were from D.J.H. [19]. *P*. *aeruginosa* PAO1 was used for flow cytometry, imaging studies (electron microscopy and laser confocal microscopy), ROS nitroblue tetrazolium assays, electrical current-exposed buffer experiments, and microarray analysis. *P*. *aeruginosa* Xen 5 was used for studies involving catalase and SOD production, antioxidant supplementation, and the lipid peroxidation TBARS assay.

### Biofilms

Biofilms were grown on Teflon or glass (for laser confocal microscopy) discs (12.5 mm x 1 mm) in 2 ml trypticase soy broth (TSB) on an orbital shaker (140 rpm) for 24 hours at 37°C in 24 well microtiter plates. *S*. *aureus* biofilms were grown in TSB supplemented with 2% glucose.

### Electrical treatment device

**Fig 1:**
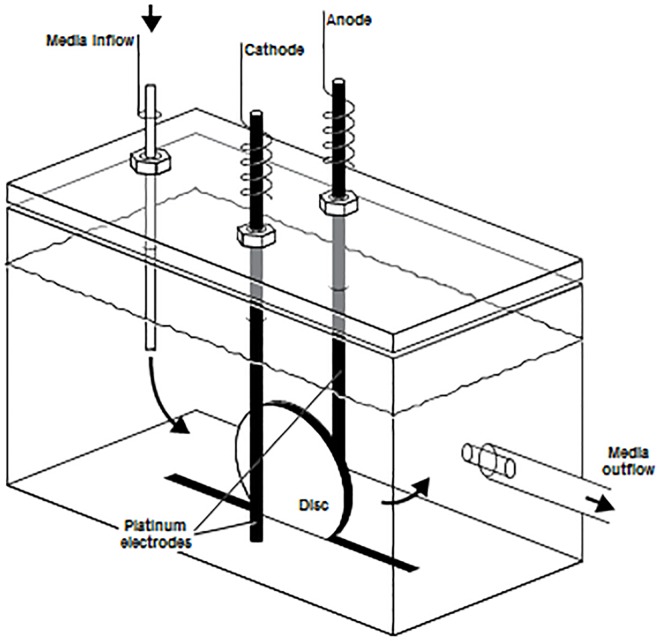
Schematic of the electrical treatment device. Electrodes were placed 3 mm away from the disc. The drawing was originally published in [20].

Designed by the Mayo Division of Engineering, the treatment device consisted of an 8-channel computer controlled current generator that delivers 20–2,000 μA of DC; alternatively, a Keithley 2400 SourceMeter (Cleveland, OH) was used. Discs containing biofilm were exposed to DC via platinum electrodes (with the exception of initial catalase, SOD, and antioxidant supplementation assays which used stainless steel electrodes) in previously-described polycarbonate test chambers [10] (Fig 1). Electrodes measured 1.5 mm in diameter and 55 mm in length. To set up each experiment, biofilm-coated discs were removed from the well plates and rinsed by gently dipping in sterile saline to remove planktonic bacteria. The discs were then placed in test chambers in an upright position with electrodes positioned on both sides, 3 mm from the disc [20]. A 1X phosphate flow buffer was prepared with 426 mg Na_2_HPO_4_, 205 mg KH_2_PO_4_, and 640 mg glucose in one liter of distilled water, adjusted to a pH of 7.4 and filter sterilized. The stock flow buffer was diluted to 3% (30 ml stock buffer + 970 ml sterile water) for each experiment. The 3% phosphate flow buffer was continuously pumped through each test chamber at 3 ml/hour.

### Quantitative culture and log reduction factor (LRF) calculations

Quantitative culture of biofilms on discs was accomplished by rinsing the discs with sterile saline, vortexing for 30 sec, sonicating for 5 min, vortexing for 30 sec, followed by serial dilution on to 5% sheep blood trypticase soy agar plates. Quantitative cultures were performed in triplicate prior to each experiment to determine initial biofilm density. Biofilm reduction was expressed using the logarithmic reduction factor (LRF), i.e., the log[(mean CFU/cm^2^ of nonexposed discs)/(mean CFU/cm^2^ of exposed discs)] [10, 21]. An initial biofilm density of ≥5 log_10_ cfu/cm^2^ was required.

### I. Visualization of cell death, and changes in bacterial morphology and biofilm architecture

#### Confocal microscopy

Following exposure to 200 or 0 μA DC for 24 hours, discs were removed from treatment chambers, gently dipped in sterile saline and transferred into 1 ml sterile saline in a 14 ml conical tube. Each disc was stained with 1.5 μl of SYTO 9 dye (3.34 mM) and propidium iodide (20 mM) using the Live/Dead BacLight Bacterial Viability kit (Molecular Probes, Eugene, OR). Discs were incubated in the dark for 15 min at room temperature and then imaged according to the manufacturer using a Zeiss LSM 510 confocal microscope (Carl Zeiss, Germany). Discs were viewed at 60X magnification, with a minimum of seven fields observed and photographed.

#### Scanning electron microscopy (SEM)

Following exposure to 200 μA DC or no current for 24 hours, discs were removed, rinsed with saline and then immediately placed into 1 ml of TRUMPS solution (4% formaldehyde, 1% glutaraldehyde in phosphate buffer, pH ~7.3). Preparation of discs for SEM was done by the Microscopy and Cell Analysis Core of the Mayo Clinic. Briefly, discs were washed twice in phosphate buffered saline, followed by two water rinses. Discs were then washed in a series of ethanol washes (30, 70, 95 and 100%), with two rinses at each concentration. Following ethanol washing, discs were placed in a critical point dryer to replace ethanol with CO_2_. Discs were removed, mounted to an aluminum stub and sputter-coated for 90 sec with gold-palladium (to enhance electrical conductivity). Discs were viewed using a Hitachi S-4700 SEM (Hitachi High Technologies America, Inc., Schaumburg, IL) at 10K magnification; a minimum of three fields were observed.

#### Flow cytometry

Following exposure to 200 μA DC or no current for 24 hours, discs were removed, dipped in sterile saline, placed into a 14 ml conical tube containing 1 ml HEPES-buffered saline, vortexed and sonicated; the resultant sonicate fluid was transferred to a microcentrifuge tube, centrifuged at 13,000 x *g* for 5 min and the supernatant removed. Bacterial pellets from three treated or control discs were combined and resuspended in 400 μl of staining mix containing 1 ml of HEPES-buffered saline and 4 μl of both component A (SYTO-10) and component B (DEAD Red; ethidium homodimer-2) from the Live/Dead Reduced Biohazard Viability/Cytotoxicity Kit (Molecular Probes). Samples were incubated in the dark at room temperature for a minimum of 15 min, and then fixed with a final concentration of 4% paraformaldehyde. Samples were transferred to the Flow Cytometry Core at the Mayo Clinic where they were analyzed on a FACSCanto flow cytometer using FACSDiva v6.1.3 software (BD Biosciences, San Jose, CA). Side scatter was used for setting a threshold, to eliminate background noise; the threshold was set at 200. SYTO-10 and DEAD Red were both excited at 488 nm wavelength and the SYTO-10 signal was collected at an emission wavelength of 530/+-30 nm, while the DEAD Red emission wavelength was collected at 585/+-42 nm. Minimal compensation was applied using single stained controls. The samples were acquired with a low sample pressure. Flow cytometry was conducted in triplicate for each organism; the figures and numbers presented are a single representative of the data.

### II. Detection of ROS and resultant enzyme production

#### ROS assay

Nitroblue tetrazolium chloride (NBT) was used to assess intracellular ROS. A stock NBT solution was prepared (10 mg/ml in water). Following exposure to 200 μA DC or no current for 5 or 10 min, discs were removed, dipped in sterile saline, and placed in tubes containing 1 mg/ml NBT. Biofilms were removed from the discs and incubated at room temperature in the dark for 30 min. To stop the reaction, 0.1M HCl was added and the tube centrifuged at 13,000 x *g* for 5 min. Biofilms from three discs were combined and resuspended in 800 μl saline, following which 400 μl of DMSO was added to release intracellular ROS. 200 μl of each sample was then placed into a well of a 96 well plate and read at 570 nm (Multiskan Plus, Thermo Scientific, Waltham, MA). Each experimental sample was read in 5 separate wells and results averaged.

#### Treatment with electrical current-exposed buffer

An experiment was set up similar to those above, with the exception that the effluent of a chamber harboring sterile discs (hereafter referred to as “electrified buffer”) was collected and pumped at a rate of 3 ml/hour for 24 hours into a second chamber containing biofilm-laden discs. Discs were aseptically removed and quantitatively cultured following exposure to buffer exposed to 2,000 μA DC for 24 hours.

#### Catalase and SOD assays

Following exposure to 200 μA DC or no current for 24 hours, chamber fluid was collected, centrifuged at 4,000 rpm and the supernatant used for the described assays. The catalase and SOD assays were conducted per the manufacturer (Cayman Chemical, Ann Arbor, MI). The catalase assay utilizes the peroxide function of catalase for determination of enzyme activity. The formaldehyde produced was measured colorimetrically at an absorbance of 540 nm using Purald as the chromogen. One unit was defined as the amount of enzyme that caused the formation of 1.0 nmol of formaldehyde/min at 25°C. The SOD assay utilizes a tetrazolium salt for detection of superoxide radicals generated by xanthine oxidase and hypoxanthine. The absorbance was read at 450 nm. One unit of SOD was defined as the amount of enzyme needed to inhibit 50% of O_2_^-^ production. Standards and buffer control were used for both assays.

#### Antioxidant supplementation assays

Biofilms were grown on discs in the presence and absence of antioxidants. Standard phosphate flow buffer or buffer supplemented with antioxidant was used. Catalase from bovine liver (Sigma, St. Louis, MO) was reconstituted in potassium phosphate buffer. The free radical scavengers D-mannitol (Sigma) and Tempol (Sigma) were reconstituted in water. Catalase was placed into the flow buffer at a final concentration of either 200 or 500 U/ml. D-mannitol was placed into the flow buffer at a final concentration of 20 or 50 mM and Tempol was placed into the flow buffer at a concentration of 1 mM. Additionally, biofilms were grown on a set of discs in TSB supplemented with 500 U/ml of catalase, 20 mM of mannitol, or 5 or 10 mM of Tempol. The discs were exposed to 20 or 200 μA DC or no current for 24 hours using a 3% phosphate buffer with or without antioxidant. The LRF from samples supplemented with antioxidants was subtracted from the LRF from samples with no antioxidant supplementation; a positive number indicates protection against the electricidal effect with a >0.5 change in the LRF considered protective.

#### *P*. *aeruginosa* mutant analyses

*P*. *aeruginosa* PAO1 and *P*. *aeruginosa* PAO1Δ*sodAB* were studied. Following exposure to 200 μA DC or no current for 24 hours, the LRF of the mutant was compared to the LRF of the parent strain.

### III. Effects of DC exposure on alternative pathways

#### Lipid peroxidation assay

The lipid peroxidation assay measuring malondialdehyde (MDA) concentration, a byproduct of lipid peroxidation and an indicator of oxidative stress, was performed using the TBARS (Thiobarbituric Acid Reactive Substances) Assay Kit (ZeptoMetrix Corporation, Buffalo, NY) according to the manufacturer’s protocol. Standards and procedures were performed according to the assay insert, with the following notes. A negative sample with 100 μl of saline was included. The assay was conducted using a 96 well black plate with an opaque bottom. 250 μl of each sample was placed into a well, and each sample was run in triplicate.

Briefly, following exposure to 200 μA DC or no current for 5 or 10 min, three discs were removed from the chambers, biofilms removed, and sonicate fluid placed into a microcentrifuge tube. Additionally, flow buffer from three current-exposed and -unexposed chambers was collected. The chamber fluid was centrifuged at 4,000 rpm for 10 min at room temperature and then resuspended in 1 ml sterile saline and transferred to a 1.5 ml microcentrifuge tube.

Microcentrifuge tubes were centrifuged at 13,000 x *g* for 5 min, and the pellet resuspended in 300 μl sterile saline. Three replicates of each were combined into one tube, leaving one tube each with cells from the exposed chamber fluid, the unexposed chamber fluid, the exposed sonicate fluid and the unexposed sonicate fluid. Tubes were centrifuged at 13,000 x *g* for 5 min and cells resuspended in 100 μl sterile saline. For the test procedure, 100 μl samples were added to 100 μl of SDS solution, along with 2.5 ml of TBA/Buffer reagent in glass tubes. Each tube was covered with a marble and incubated for 60 min at 95°C, cooled in an ice bath for 10 min, and centrifuged at 3000 rpm for 15 min. The supernatant was removed for analysis, by placing 250 μl into three wells for each sample. Fluorescence was read in a Fluoroskan Ascent™ Microplate Fluorometer (Thermo Scientific) with excitation at 530 nm and emission at 550 nm. The MDA concentration was measured for three replicates for each sample. All values were normalized against the log_10_ CFU/cm^2^ for each sample.

### IV. Transcriptional profiling

#### Transcriptional profiling

Microarray analysis was performed using *P*. *aeruginosa* PAO1. Three discs each were exposed to 200 μA DC or no current for 60 min. Discs were then removed, placed into 1 ml of sterile saline, biofilms removed, and the sonicate fluid from the three discs combined for both exposed and unexposed samples. Samples were centrifuged at 4,000 rpm for 5 min and supernatant discarded. Bacteria were resuspended in 500 μl saline, 1 ml of RNAprotect (Qiagen, Valencia, CA) added and samples incubated at room temperature for 5 min, followed by centrifugation at 4,000 rpm for 10 min. To isolate RNA from the bacterial samples, the RNeasy kit (Qiagen) was used; RNA was extracted per the package insert, with the addition of RNase free DNase treatment (Qiagen). RNA was eluted by adding 40 μl of RNase-free water and centrifuging at 14,000 rpm for 1 min. RNA was quantitated using the Qubit RNA-HS assay (Life Technologies, Carlsbad, CA). Samples were submitted to the Medical Genome Facility at Mayo Clinic. Microarray analysis was conducted according to manufacturer’s instructions for the Affymetrix SensationPlus FFPE Amplification and 3’ IVT Labeling kit. 3.9 μg of labeled product was added to a hybridization solution prepared according to manufacturer’s instructions (Affymetrix, Santa Clara, CA). Labeling controls (Lys, Dap, Phe, Thr), hybridization controls (BioB, BioC, BioD, and Cre), and oligonucleotide B2 were spiked into the labeling and hybridization reactions, respectively, to ensure quality of the procedure. The hybridization solution was heated at 99°C for 5 min, followed by incubation at 45°C for 5 min and then centrifuged briefly at high speed before applying the sample onto Affymetrix *P*. *aeruginosa* arrays®. Hybridization was performed at 50°C for 16 hours in a rotisserie oven at 60 rpm. The solutions were removed, and arrays washed and stained as described in the GeneChip® Expression Analysis Technical Manual (Affymetrix). The hybridized arrays were scanned using a GeneChip® 3000 scanner and Affymetrix GeneChip® Command Console software v.4.0 to quantitatively analyze the scanned image. All control parameters were confirmed to be within normal ranges.

**Pre-processing and normalization of microarray data:** Transcriptome profiles were measured using Affymetrix Whole Genome *Pseudomonas aeruginosa* GeneChip arrays. Pre-processing and normalization was done using the Partek microarray data analysis software (Partek, St. Louis, MO). Partek pre-processes raw intensity files from microarray experiments using the robust multiarray algorithm’s (RMA’s) background subtraction and uses quantile normalization as the normalization technique.

**Differential gene expression analysis:** The samples from different groups were compared using standard t-tests. Significantly changed genes were prioritized using fold change measures. Cut-offs for fold changes at 1.5-, 2- and 3-fold increase or decrease were applied.

### Statistics

Differences in the LRF between DC exposed and unexposed groups were compared using Wilcoxon rank sum test; the test was 2 sided, with p-values less than 0.05 considered statistically significant. In *P*. *aeruginosa* mutant studies, differences in the LRF between wild-type and mutants were compared using the Wilcoxon rank sum test.

## Results

### I. Visualization of cell death, and changes in bacterial morphology and biofilm architecture

#### Cell death and changes in biofilm architecture visualized through confocal microscopy and SEM

**Fig 2:**
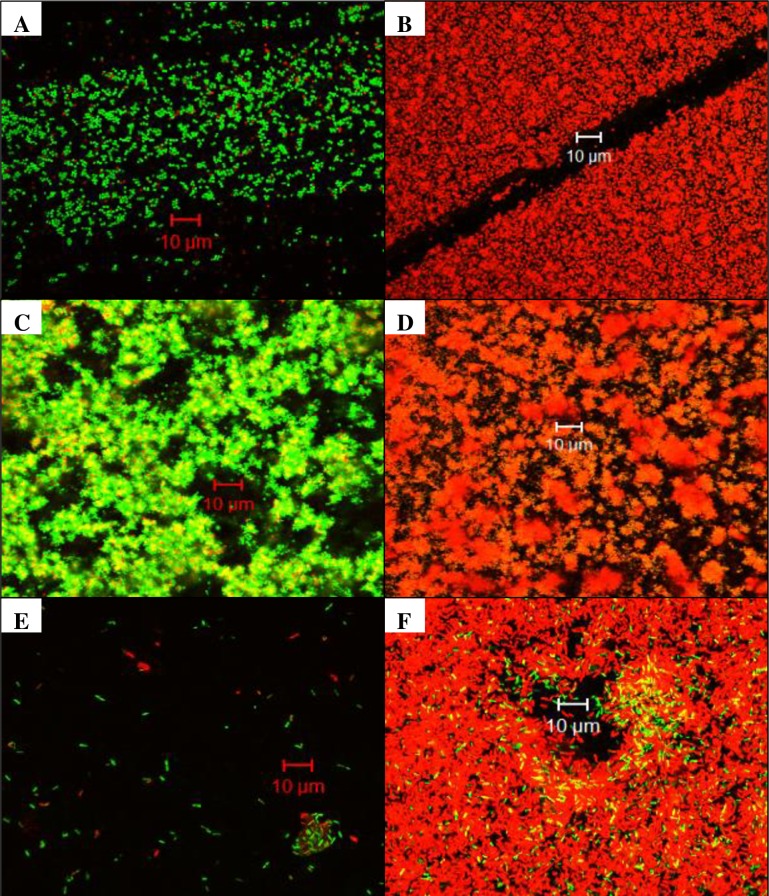
Confocal microscopy of biofilms after 24 hours of no exposure (left) or exposure (right) to direct current (DC). *S*. *aureus* control (**A**) and 200 μA DC (**B**); *S*. *epidermidis* control (**C**) and 200 μA DC (**D**); *P*. *aeruginosa* control (**E**) and 200 μA DC **(F)**. All images were taken at 60X magnification; a minimum of seven fields were observed. A representative field for each bacterial biofilm sample is shown.

When examined by confocal microscopy, all bacteria studied showed an increase in propidium iodide staining, indicating cell death (Fig 2). Compared to controls, both *S*. *aureus* (Fig 2A and 2B) and *P*. *aeruginosa* (Fig 2E and 2F) exposed to DC had an apparent increased amount of cells visible on the disc, although the majority of them were dead. *S*. *epidermidis* biofilms exhibited a ruffled edge and increased cell death when exposed to DC (Fig 2D) compared to controls (Fig 2C). Increased aggregation of cells was common for all bacterial biofilms exposed to DC. Quantitative cultures of all bacteria showed a decrease in viable bacteria following DC exposure when compared with control discs (Table 1).

**Table 1:** Mean bacterial biofilm quantities and LRF values at 24 hours (n = 3 for all samples).

Organism	Direct Current	log_10_ cfu/cm^2^	Std. Dev.	LRF
***S*. *aureus***	None	5.37	0.15	**2.20**
	200 μA	3.17	0.71	
***S*. *epidermidis***	None	7.09	0.19	**3.20**
	200 μA	3.89	0.75	
***P*. *aeruginosa***	None	6.92	0.31	**2.04**
	200 μA	4.88	0.35	

The mean bacterial quantities were calculated following exposure to either no current or 200 μA DC after 24 hours (n = 3 for all samples). Std. Dev., standard deviation.

**Fig 3:**
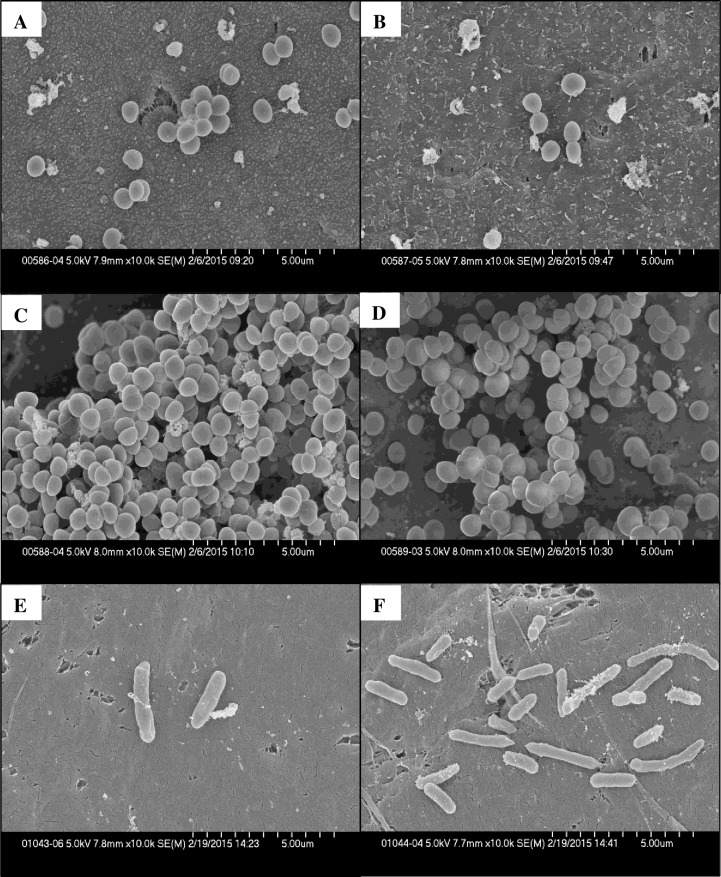
Scanning electron micrographs of biofilm-laden discs exposed to 200 μA direct current (DC) or no exposure for 24 hours. *S*. *aureus* control (**A**) or 200 μA DC (**B**); *S*. *epidermidis* control (**C**) or 200 μA DC **D**); *P*. *aeruginosa* control (**E**) or 200 μA DC (**F**). All images were taken at 10K magnification and a minimum of three fields were observed. A representative field for each bacterial biofilm sample is shown.

SEM was performed on *S*. *aureus*, *S*. *epidermidis* and *P*. *aeruginosa* biofilms (Fig 3). All biofilms demonstrated changes as a result of electrical exposure. There was an increase in cell debris in DC-exposed *S*. *aureus* biofilms (Fig 3B) compared to control biofilms (Fig 3A). *S*. *epidermidis* biofilms exposed to DC contained organisms that had apparent defects in cell division as evidenced by an incomplete division septum and clumping of organisms (Fig 3D) compared with controls (Fig 3C). *P*. *aeruginosa* biofilm cells exposed to DC exhibited elongation and chaining (Fig 3F) compared with controls (Fig 3E). Quantitative cultures done of discs not used for SEM for all bacteria showed a decrease in viable bacteria when compared with controls (Table 2).

**Table 2:** Mean bacterial biofilm quantities and LRF values.

Organism	Direct Current	log_10_ cfu/cm^2^	Std. Dev.	LRF
***S*. *aureus***	None	5.88	0.45	**1.20**
	200 μA	4.68	0.78	
***S*. *epidermidis***	None	6.66	0.12	**4.83**
	200 μA	1.83	1.57	
***P*. *aeruginosa***	None	5.85	0.04	**3.49**
	200 μA	2.36	1.04	

The mean bacterial quantities were calculated following exposure to either no current or 200 μA DC after 24 hours (n = 3 for all samples). Std. Dev., standard deviation.

#### Cell death was confirmed using flow cytometry

**Fig 4:**
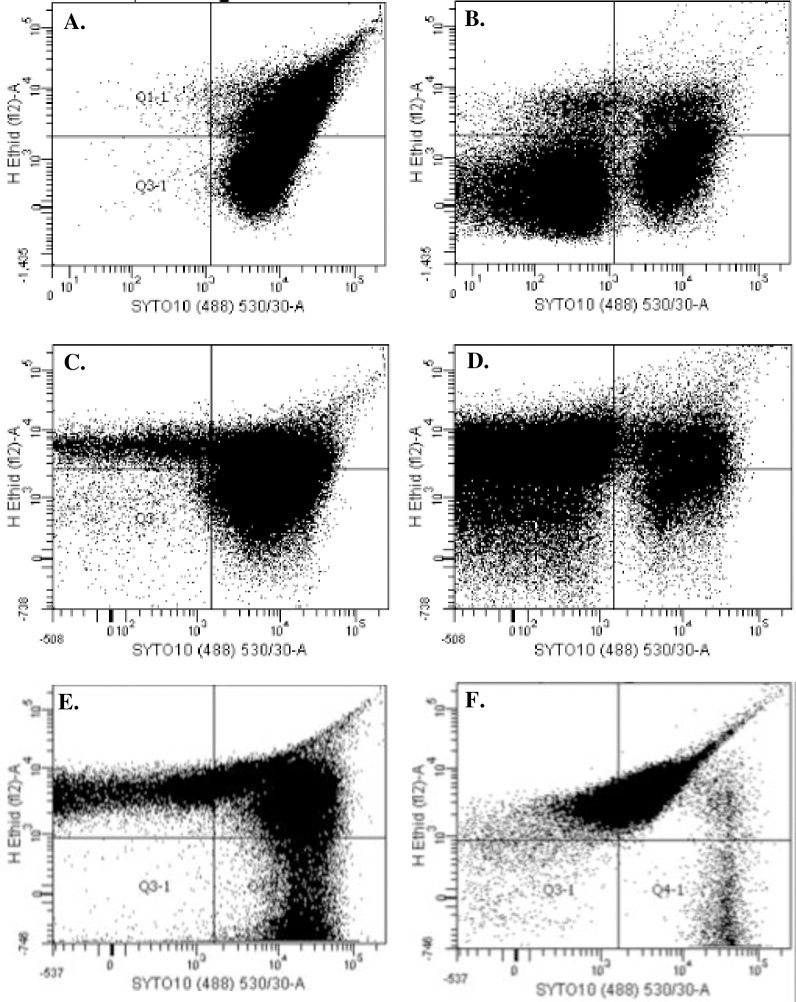
Flow cytometric analysis of biofilms exposed to 200 μA direct current (DC) for 24 hours, and controls. *S*. *aureus* control (**A**) and DC exposure (**B**); *S*. *epidermidis* control (**C**) and DC exposure (**D**); and *P*. *aeruginosa* control (**E**) and DC exposure (**F**). Experiments were performed in triplicate for each organism; a representative graph is shown for each bacterium.

Flow cytometry was performed on *S*. *aureus*, *S*. *epidermidis* and *P*. *aeruginosa*. All bacterial strains tested exhibit a shift from SYTO-10 staining (live, Q 4–1) in controls to DEAD-Red staining (dead, Q 1–1) in bacteria exposed to DC (Fig 4). *S*. *aureus* biofilms showed a shift from 68% live bacteria in control samples (Fig 4A) to 22% live bacteria in DC-exposed samples (Fig 4B). *S*. *epidermidis* biofilms demonstrated a shift from 66% live bacteria in controls samples (Fig 4C) to 10% live bacteria in DC-exposed samples (Fig 4D). *P*. *aeruginosa* biofilms demonstrated a shift from 26% live bacteria in control samples (Fig 4E) to 1% live bacteria in DC-exposed samples (Fig 4F).

### II. Detection of ROS and resultant enzyme production

#### Exposure to electricity causes an increase in intracellular ROS

**Fig 5:**
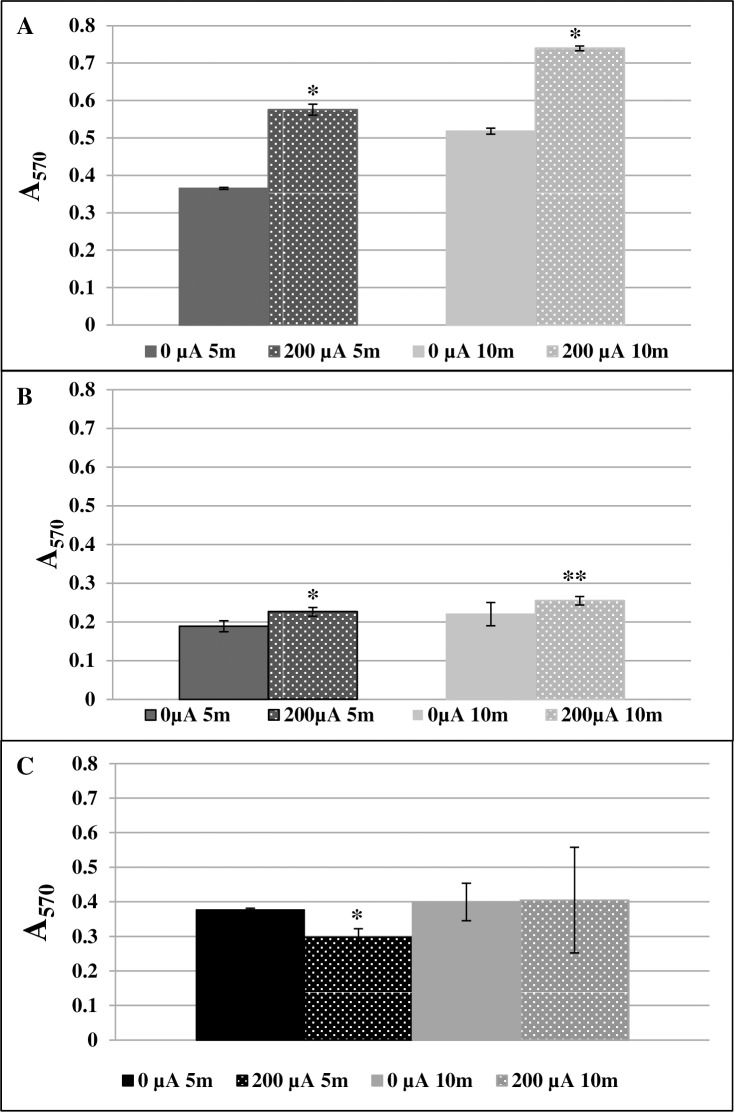
Detection of reactive oxygen species (ROS) using nitroblue tetrazolium. Samples not exposed to current (shown as 0 μA) were compared with those exposed to 200 μA direct current (DC) after 5 and 10 minutes. **A.** ROS production in *S*. *aureus* *p = 0.0088; **B.** *S*. *epidermidis* *p = 0.0088 **p = 0.012; and **C.** *P*. *aeruginosa* *p = 0.009, compared with control.

Measurement of intracellular ROS using nitroblue tetrazolium chloride (NBT) was performed on *S*. *aureus*, *S*. *epidermidis* and *P*. *aeruginosa* biofilms. NBT precipitation was increased for *S*. *aureus* biofilms that had been exposed to DC for both 5 and 10 min (p = 0.0088 for both time points) (Fig 5A), and for *S*. *epidermidis* biofilms exposed to DC for both 5 and 10 min (p = 0.0088 and p = 0.012, respectively) (Fig 5B), indicating increased production of intracellular ROS following DC exposure. *P*. *aeruginosa* biofilms had decreased levels of precipitated NBT at 5 min (p = 0.009), but not at 10 min (p = 0.117) (Fig 5C).

#### Effects of exposure to electrified buffer

**Fig 6:**
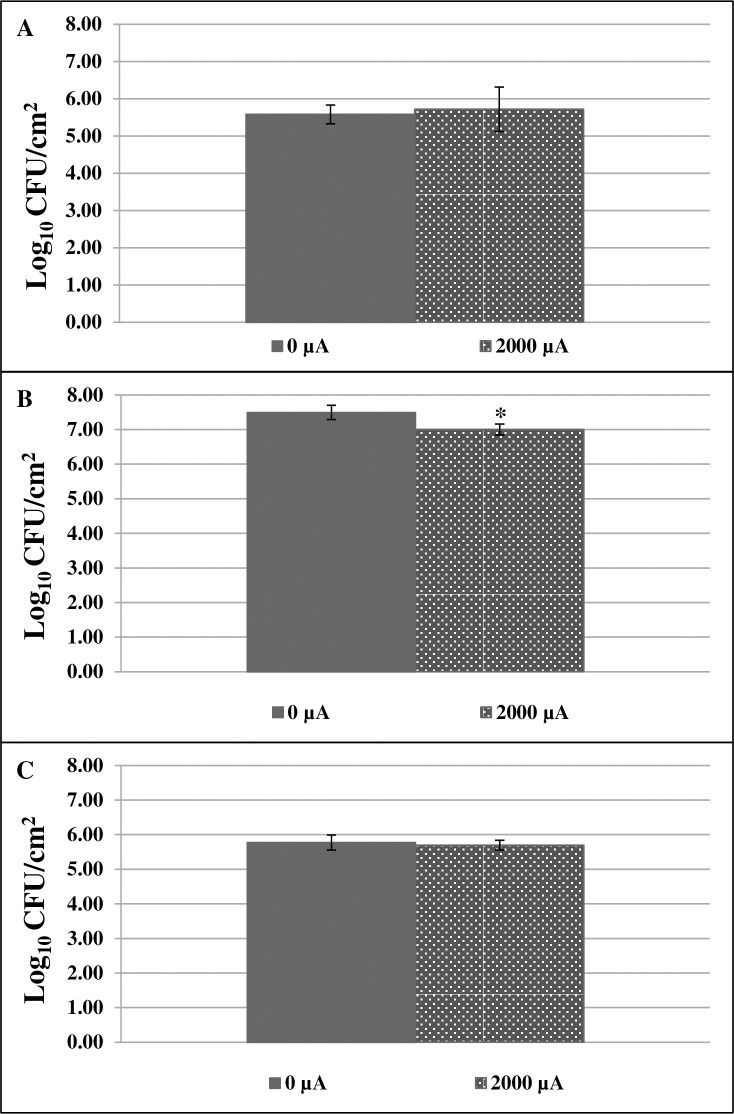
Pre-treating buffer with 2,000 μA direct current (DC) did not affect *S*. *aureus* and *P*. *aeruginosa* and slightly affected *S*. *epidermidis* over a period of 24 hours. **A.** *S*. *aureus* biofilms **B.** *S*. *epidermidis* biofilms *p = 0.0463; **C.** *P*. *aeruginosa* biofilms. Samples not exposed to DC are shown as 0 μA.

Experiments were performed using *S*. *aureus*, *S*. *epidermidis* or *P*. *aeruginosa* biofilms. For *S*. *aureus* and *P*. *aeruginosa*, there was no decrease in biofilm on discs exposed to electrified buffer, compared to those exposed to standard buffer (Fig 6A and 6C). For *S*. *epidermidis*, however, there was a decrease in biofilm on discs exposed to electrified buffer, compared to those exposed to standard buffer (p = 0.0495) (Fig 6B).

#### Exposure to electricity leads to increased catalase and SOD production

Catalase and SOD production in response to ROS were measured using *S*. *aureus*, *S*. *epidermidis* and *P*. *aeruginosa* biofilms. Both catalase and SOD activity were increased for biofilms of all three organisms tested following exposure to DC. The mean catalase activity of *S*. *aureus* biofilms increased from 0 nmol/min/ml for control samples to 20.04 nmol/min/ml for samples exposed to DC. For *S*. *epidermidis* biofilms, the mean catalase activity increased from 0.96 nmol/min/ml for control samples to 26.27 nmol/min/ml for samples exposed to DC, and for *P*. *aeruginosa* biofilms, the mean catalase activity increased from 0.17 for control samples to 16.59 for samples exposed to DC (Table 3). This correlates to a 20-fold, 27-fold, and 98-fold increase following DC exposure for the three organisms’ biofilms, respectively. An increase in SOD activity was measured with a mean of 0 units/ml for all three isolates not exposed to DC, and 1.02 units/ml for *S*. *aureus* (p = 0.004) (n = 5), 1.52 units/ml for *S*. *epidermidis* (p = 0.0021) (n = 6), and 0.76 units/ml for *P*. *aeruginosa* (p = 0.0019) (n = 6) biofilms exposed to DC (Table 3).

**Table 3:** Concentrations of catalase and superoxide dismutase increase in response to DC.

Organism	Catalase(nmol/min/ml)				Superoxide dismutase(units/ml)				
	0 μA	Std. Dev.	200 μA	Std. Dev.	0 μA	Std. Dev.	200 μA	Std. Dev.	P value
***S*. *aureus***	0	0	20.04	3.06	0	0	1.02	0.27	0.004
***S*. *epidermidis***	0.96	0.43	26.27	1.13	0	0	1.52	0.26	0.0021
***P*. *aeruginosa***	0.17	0.25	16.59	2.79	0	0	0.76	0.11	0.0019

Mean concentration of catalase and superoxide dismutase produced after 24 hours of exposure to200 μA direct current. For catalase assay; n = 2, for SOD assay; n = 6 for *S*. *epidermidis* and *P*. *aeruginosa*, n = 5 for *S*. *aureus*. Samples not exposed to DC are shown as 0 μA. Std. Dev., standard deviation.

#### Supplementation with catalase, D-mannitol and Tempol protects bacterial biofilms from the electricidal effect

*S*. *aureus*, *S*. *epidermidis* and *P*. *aeruginosa* biofilms were supplemented with catalase, D-mannitol or Tempol. Table 4 summarizes differences in the LRF observed in samples supplemented with various antioxidants compared to those with no antioxidant. The addition of catalase during biofilm growth resulted in >0.5 log_10_ cfu/cm^2^ protection for all three isolates’ biofilms. The addition of 200 U/ml of catalase in the flow buffer resulted in >0.5 LRF protection for both *S*. *aureus* and *S*. *epidermidis* biofilms, while the addition of 500 U/ml catalase in the flow buffer provided a >0.5 LRF protection for biofilms formed by all three isolates. Significant protection using catalase was achieved for *S*. *aureus* biofilms (p = 0.0463 in all three scenarios) and there was a trend toward protection for *S*. *epidermidis* biofilms. The addition of the free radical scavenger D-mannitol (50 mM) to the flow buffer resulted in >0.50 LRF protection of *S*. *epidermidis* and *P*. *aeruginosa* biofilms. The addition of the free radical scavenger Tempol (10 mM in biofilm/1 mM in buffer) protected against the electricidal effect for *S*. *epidermidis* biofilms exposed to 200 μA DC (p = 0.0495) and *P*. *aeruginosa* biofilms exposed to 20 μA DC (p = 0.0495).

**Table 4:** Supplementation of bacterial biofilms with catalase, D-mannitol or Tempol protects against the electricidal effect.

Antioxidant	Concentration of Antioxidant	*S*. *aureus* ΔLRF	*S*. *epidermidis* ΔLRF	*P*. *aeruginosa* ΔLRF
**Catalase 200 μA**	500 U/ml in biofilm	0.75*	**0.62**	**0.79**
	200 U/ml in buffer	0.85*	**0.82**	0.3
	500 U/ml in buffer	1.18*	**1.1**	**0.82**
**Mannitol 200 μA**	20 mM in biofilm	0.28	0.09	-0.29
	20 mM in buffer	-0.82	0.47	**0.79**
	50 mM in buffer	-0.78	**0.58**	**0.8**
**Tempol 200 μA**	5mM in biofilm/1 mM in buffer	-0.42	0.32	0.2
	10mM in biofilm/1 mM in buffer	0.12	1.59**	0.33
**Tempol 20 μA**	5mM in biofilm/1 mM in buffer	ND	ND	**0.95**
	10mM in biofilm/1 mM in buffer	ND	ND	2.10**

Bacterial biofilms were exposed to 200 μA direct current. A positive number indicates protection; bold font indicates a greater than 0.5 decrease in LRF. ND = Not determined

*p = 0.0463

**p = 0.0495, n = 3 for all samples.

#### Enhanced electricidal effect against a *P*. *aeruginosa* SOD mutant

**Fig 7:**
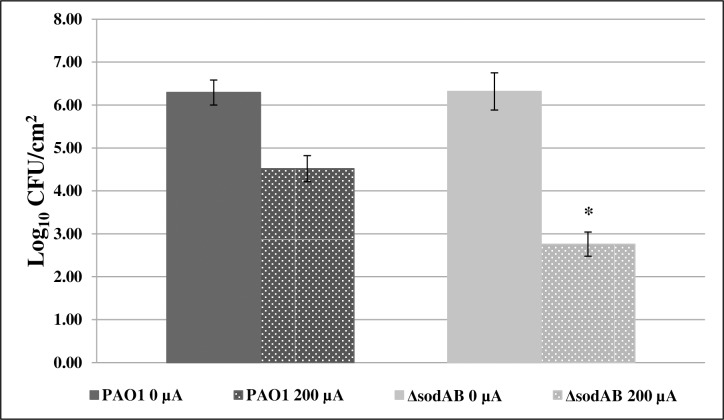
The electricidal effect is enhanced in PAO1Δ*sodAB*. *P*. *aeruginosa* PAO1Δ*sodAB* compared with parental control following exposure to 200 μA direct current (DC) for 24 hours, *p = 0.0495, n = 3 for all samples. Samples not exposed to DC are shown as 0 μA.

Comparing the LRFs of *P*. *aeruginosa* PAO1 and *P*. *aeruginosa* PAO1Δ*sodAB* following exposure to 200 μA DC after 24 hours, Δ*sodAB* showed enhanced susceptibility to electricity exposure compared with the parent strain (p = 0.0495). *P*. *aeruginosa* PAO1 had an LRF of 1.77 while *P*. *aeruginosa* PAO1*ΔsodAB* had an LRF of 3.56 (Fig 7).

### III. Effects of DC exposure on alternative pathways

#### Lipid peroxidation in response to electricity exposure

**Fig 8:**
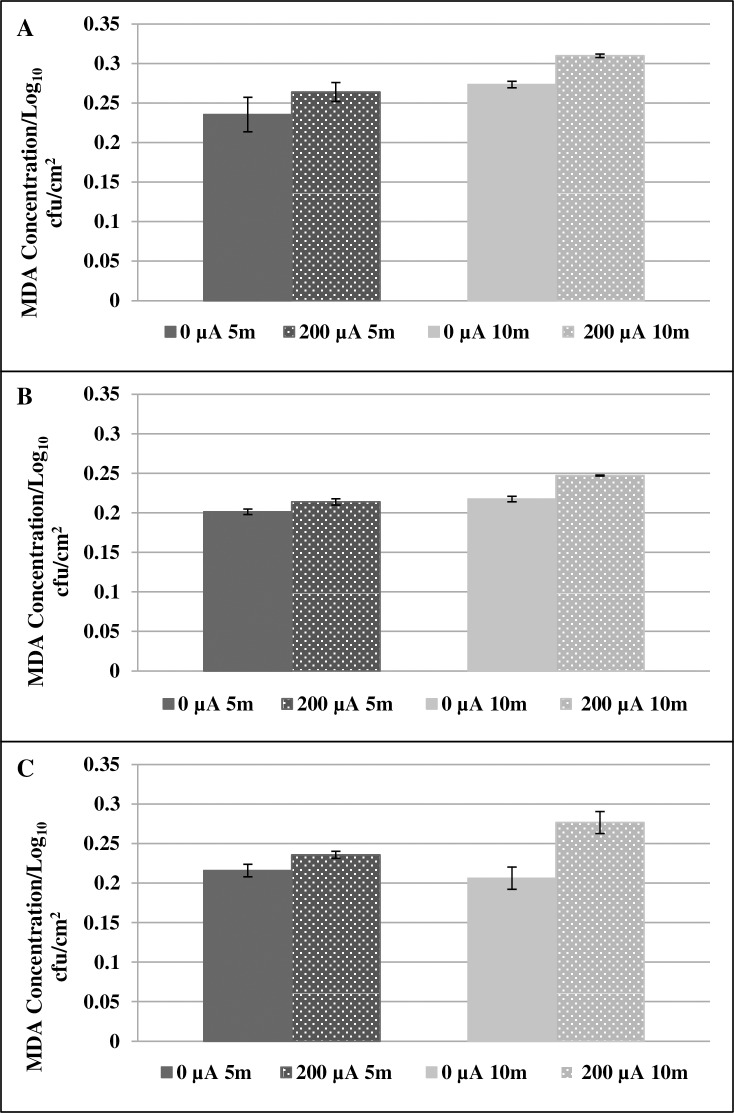
Lipid peroxidation in response to DC. Lipid peroxidation measured by MDA production in *S*. *aureus* (**A**), *S*. *epidermidis* (**B**) and *P*. *aeruginosa* (**C**) following no exposure (shown as 0 μA) or exposure to 200 μA direct current for 5 or 10 minutes. Samples were read in triplicate and normalized to the log_10_ cfu/cm^2^.

Lipid peroxidation as an indicator of oxidative stress was measured using *S*. *aureus*, *S*. *epidermidis* and *P*. *aeruginosa* biofilms. Comparing samples exposed to DC with their unexposed counterparts, a non-statistically significant trend toward increased lipid peroxidation following DC exposure was measured with an increase in MDA concentration for the three isolates (p = 0.121) (Fig 8).

### IV. Transcriptional profiling by microarray analysis

#### Transcriptional profiling showed transcripts related to stress responses/cell death following exposure to DC in *P*. *aeruginosa*

Microarray analysis showed several transcripts to be up- and down-regulated following DC exposure. Transcripts that were up- or down-regulated three fold or more were reviewed (Table 5). Of particular interest was the increase in transcripts encoding the two-component regulatory system PhoR/B, the transcriptional regulator PsrA, the organic hydroperoxide resistance protein Ohr, the porin precursor protein OprO, PchA and PchG (proteins involved in the siderophore pyochelin) and PvdN, a protein involved in the formation of the siderophore pyoverdin. Transcripts of interest that were decreased in current-exposed biofilms encoded for proteins involved in flagella formation (FlgC, FlgD, FlgE, FlgF, FlgK and FlgL), heat shock proteins (HtpG, HslV and GrpE), anti-sigma factor MucA, and genes involved in metabolism such as xanthine dehydrogenase, glycosyl and glutamine transferases, glycerol kinase and arginine deaminase, as well as subunits of cytochrome c oxidase. The data discussed in this publication have been deposited in NCBI's Gene Expression Omnibus and can be found under accession number GSE90757 [22].

**Table 5:** Transcript changes in *P*. *aeruginosa* PAO1 in response to direct current.

**Up-regulated transcripts following exposure to direct current**		
**Probeset ID**	**Gene Symbol**	**Gene Title**
PA5361_phoR_at	*phoR*	two-component sensor PhoR
PA5360_phoB_at	*phoB*	two-component response regulator PhoB
PA3006_at	*psrA*	transcriptional regulator PsrA
PA0283_sbp_at	*sbp*	sulfate-binding protein precursor
PA4231_pchA_at	*pchA*	salicylate biosynthesis isochorismate synthase
PA3280_oprO_at	*oprO*	Pyrophosphate-specific outer membrane porin OprO precursor
PA4224_at	*pchG*	pyochelin biosynthetic protein PchG
PA2394_at	*pvdN*	PvdN
PA2850_ohr_at	*ohr*	organic hydroperoxide resistance protein
PA4205_at	*mexG*	hypothetical protein
PA4226_pchE_at	*pchE*	dihydroaeruginoic acid synthetase
PA3221_csaA_at	*csaA*	CsaA protein
PA1927_metE_at	*metE*	5-methyltetrahydropteroyltriglutamate-homocysteine S-methyltransferase
**Down-regulated transcripts following exposure to direct current**		
**Probeset ID**	**Gene Symbol**	**Gene Title**
PA1523_xdhB_at	*xdhB*	xanthine dehydrogenase
PA4893_ureG_at	*ureG*	urease accessory protein UreG
PA1456_cheY_at	*cheY*	two-component response regulator CheY
PA2231_at	*pslA*	probable glycosyl transferase
PA0297_at	*spuA*	probable glutamine amidotransferase
PA5172_arcB_at	*arcB*	ornithine carbamoyltransferase, catabolic
PA4464_ptsN_at	*ptsN*	nitrogen regulatory IIA protein
PA1175_napD_at	*napD*	NapD protein of periplasmic nitrate reductase
PA2862_lipA_at	*lipA*	lactonizing lipase precursor
PA1596_htpG_at	*htpG*	heat shock protein HtpG
PA5053_hslV_at	*hslV*	heat shock protein HslV
PA4762_grpE_at	*grpE*	heat shock protein GrpE
PA3581_glpF_at	*glpF*	glycerol uptake facilitator protein
PA3582_glpK_at	*glpK*	glycerol kinase
PA4812_fdnG_at	*fdnG*	formate dehydrogenase-O, major subunit
PA1087_flgL_at	*flgL*	flagellar hook-associated protein type 3 FlgL
PA1086_flgK_at	*flgK*	flagellar hook-associated protein 1 FlgK
PA1080_flgE_at	*flgE*	flagellar hook protein FlgE
PA1081_flgF_at	*flgF*	flagellar basal-body rod protein FlgF
PA1078_flgC_at	*flgC*	flagellar basal-body rod protein FlgC
PA1079_flgD_at	*flgD*	flagellar basal-body rod modification protein FlgD
PA4024_eutB_at	*eutB*	ethanolamine ammonia-lyase large subunit
PA0105_coxB_at	*coxB*	cytochrome c oxidase, subunit II
PA0106_coxA_at	*coxA*	cytochrome c oxidase, subunit I
PA5171_arcA_at	*arcA*	arginine deiminase
PA0763_mucA_at	*mucA*	anti-sigma factor MucA

*P*. *aeruginosa* PAO1 transcripts increased or decreased three-fold or more in any of the four replicates in biofilms exposed to 200 μA DC for 60 minutes.

## Discussion

Results of this study show that decreases in bacterial quantities after exposure to 200 μA DC are primarily due to death of the bacteria within biofilms. Additionally, production of ROS appears to be at least partly responsible for cell death due to DC. Confocal microscopy showed that for all strains studied, the application of DC led to an increase in cell death (Fig 2). This observation was supported by flow cytometric data showing a shift from live staining in control samples to dead staining in DC-exposed samples (Fig 4). Notably, *S*. *aureus* biofilms exposed to DC had a dramatic increase in the percentage of bacteria that was unstained (69%). We hypothesize that this occurred because the cells were damaged, leading to cell lysis and loss of nucleic acid. Loss of nucleic acid would be expected to affect the ability of nucleic acid stains to bind in the cells, leading to decreased fluorescent signal [23, 24]. Additionally, *P*. *aeruginosa* biofilms had a large proportion of bacteria that was dually stained. Previous work has shown that SYTO stains do not penetrate Gram-negative bacteria well because of their cell envelope structure [24], possibly explaining the findings. However, we observed a shift to dead stained bacteria in the samples exposed to DC when compared to unexposed samples. The confocal microscopy and flow cytometry data together support our hypothesis that the decrease in bacterial titers in response to DC is due to an increase in cell death, and not just detachment from the disc surface.

We had hypothesized that the decrease in bacteria recovered from discs following DC exposure was due to an increase in ROS production induced by electrical current. Using a colorimetric NBT assay, we detected an increase in intracellular ROS in both *S*. *aureus* and *S*. *epidermidis* biofilms after 5 and 10 min of exposure to DC and a decrease in *P*. *aeruginosa* biofilms exposed to 200 μA DC after 5 min (Fig 5). We also observed that exposing biofilms to buffer that was previously exposed to 2,000 μA DC did not markedly affect bacterial biofilms (Fig 6), suggesting that bacterial death is, in part, mediated by products that are directly produced by bacteria in response to DC. Another way to determine if ROS play a role in bacterial death was to measure the production of key enzymes and to determine the effect of antioxidant supplementation. The production of catalase and SOD was increased in *S*. *aureus*, *S*. *epidermidis* and *P*. *aeruginosa* biofilms exposed to DC (Table 3). Supplementing both biofilms and flow buffer with catalase prevented a loss of biofilm bacteria for *S*. *aureus* but not the other organisms tested (p = 0.0463) (Table 4). Supplementation with Tempol (10mM in biofilm/1mM in buffer) increased survival of *S*. *epidermidis* and *P*. *aeruginosa* biofilms exposed to DC (Table 4).

We sought to determine if products produced in the buffer during exposure to electrical current were sufficient to induce killing on their own (Fig 6). *S*. *aureus* and *P*. *aeruginosa* biofilms exposed to buffer that had previously been exposed to electrical current did not have a decrease in bacterial quantities. *S*. *epidermidis* biofilms did decrease statistically after exposure to this buffer (p = 0.0463), however, this data likely does not indicate that the electrified buffer is sufficient to decrease biofilms since the bacterial quantity found on the disc treated with electrified buffer was high (7 log_10_ CFU/cm^2^), and the observed statistical significance likely relates to the very small standard deviation in both the DC exposed and untreated biofilms.

*P*. *aeruginosa* PAO1Δ*sodAB* exhibited greater susceptibility to 200 μA DC than did its parental strain (Fig 7), suggesting that by removing SOD production, bacteria are less able to remove toxic oxygen species produced in response to DC. This result further strengthens our hypothesis that bacterial death mediated by DC is at least partially dependent upon an increase in ROS.

We observed a trend toward increased lipid peroxidation following DC exposure (Fig 8). While we did measure increases catalase and SOD in response to ROS following 24 hours of exposure to DC, other markers of oxidative stress were challenging to detect. We performed the lipid peroxidation and ROS production assays after a short treatment of duration, as after 24 hours there were not enough bacteria remaining on the discs to perform the assays. We tested 5 and 10 min because we have previously observed that H_2_O_2_ is detectable in the chamber fluid at those times. A potential reason for the lack of ROS in samples exposed to DC may be that they are dissipating too quickly to be detected using the specific assays. Another possible reason for the difficulty in assessing ROS and lipid peroxidation is that the NBT and TBARS assays are not ideal for prokaryotic organisms.

Transcripts encoding proteins for the PhoRB two-component system were increased in *P*. *aeruginosa* biofilms exposed to DC. This regulatory system responds to conditions where P_i_ is limited by up- or down-regulating target genes [25]. While this regulon plays a role in the up- and down-regulation of a large number of genes, it contributes to both the virulence and the survival of the organism under stringent conditions [26]. Although up-regulation may be due to the limited phosphate in the buffer, comparisons were done against control biofilms receiving no DC, leading us to believe that additional stress is being applied to the treated biofilms. Additionally, an increase in the phosphate-specific porin OprO transcript suggests that bacteria are actively trying to transport phosphate during exposure to DC [27]. It has also been proposed that under P_i_ limiting conditions, catalase is induced by *P*. *aeruginosa* [28], providing an explanation for the increase in catalase observed herein. The induction of the transcript encoding for *ohr* suggests that toxic hydroperoxidases are produced in the bacteria in response to DC exposure. This protein’s function is to convert hydroperoxidases to less toxic metabolites [29]. *P*. *aeruginosa* exposed to DC increased transcription of genes responsible for the formation of the pyochelin (i.e., *pchA* and *pchG*) and pyoverdin (*pvdN*) siderophores, which are released into the extracellular environment to bind iron under iron-limiting growth conditions and [30–33].

Transcripts encoding structural flagellar proteins, specifically FlgE, FlgK and FlgL, secretion substrates of the injectisome type three secretion system (T3SS) which serve as antagonists to the host-defense inflammasome, were decreased in treated *P*. *aeruginosa* [34]. Flagellar proteins are involved in motility; their expression is likely decreased in a biofilm state in order to stabilize the biofilm [35]. The further decrease of flagellar proteins in *P*. *aeruginosa* exposed to DC compared with unexposed biofilms may be a result of the bacteria further attempting to stabilize the biofilm under stress.

Genes encoding heat shock proteins HtpG, HslV and GrpE were decreased in biofilms exposed to DC compared to unexposed biofilms. These proteins are expressed in response to a number of environmental stresses, including nutrient limitation, exposure to harmful chemicals and changes in pH [36–38], and also play a role in the secretion of exopolysaccharides [39]. It is possible that cells exposed to DC are unable to mount a proper stress response, thereby leading to their death/dispersal from the biofilm.

We show that the decrease in bacterial quantities due to electrical current exposure is due to cell death and not primarily detachment from the material surface. The data presented also suggest that the formation of ROS, measured through the increase in SOD and catalase, may contribute to bacterial death in response to DC exposure. Previous studies using *S*. *epidermidis* and similar treatment devices with flow media containing NaCl, showed a decrease in bacterial quantities ascribed to the production of hypochlorous acid [40]. Although our study used a flow medium that did not contain NaCl, it is possible that small amounts of NaCl entrained with the biofilms, may have resulted in the production of hypochlorous acid herein.

Additionally, differences between bacterial species have been observed; primarily between Gram-negative *P*. *aeruginosa* and the Gram-positives *S*. *aureus* and *S*. *epidermidis*. Differences may be due to differences in cell wall structure between the two types of organisms. The outer membrane of *P*. *aeruginosa* may be affected differently by the electrical current, thus leading to a different response at a genetic level. Differences in the flow cytometry data between *P*. *aeruginosa* and the two *Staphylococcus* species studied may be due to differential staining of *P*. *aeruginosa* [41]. Limitations of this study include the limited resources available to monitor ROS production in bacteria, the instability of ROS once produced and the decrease in the amount of live organisms present after exposure to DC (limiting our ability to study them). Additionally, in future studies, it would be beneficial to perform a transcriptional analysis of Gram-positive organisms, including *S*. *aureus* and *S*. *epidermidis*, because Gram-negative bacteria such as *P*. *aeruginosa* may have different responses to electrical current than Gram-positive bacteria. *P*. *aeruginosa* PAO1 was chosen because a suitable microarray was commercially available.

Prior to this work, we had observed a decrease in bacterial biofilms recovered from Teflon discs following exposure to DC; however it was unclear what was mediating the decrease, whether detachment or cell death. Through the experiments outlined herein, we determined that cell death occurs in response to DC, likely mediated, at least in part, by an increase in ROS leading to stress on the cells and ultimately, cell death. The trend toward an increase in lipid peroxidation and the gene transcripts that were altered in response to DC also support our hypothesis that cell death is due to stress responses following exposure to DC.
